# Improved sand cat swarm optimization algorithm assisted GraphSAGE-GRU for remaining useful life of engine

**DOI:** 10.1038/s41598-025-91418-w

**Published:** 2025-02-26

**Authors:** Yongliang Yuan, Ruifang Li, Guohu Wang, Xiaojing Lv

**Affiliations:** 1 School of Mechanical and Electrical Engineering, Zhengzhou University of Industry Technology, Zhengzhou, China; 2 Present Address: School of Mechanical and Power Engineering, Henan Polytechnic University, Jiaozuo, China

**Keywords:** Remaining useful life, Aero-engine, Graph SAmple and aggreGatE, Gated recurrent unit, Sand cat swarm optimization algorithm, Mechanical engineering, Computer science

## Abstract

With the development of deep learning, the potential for its use in remaining useful life (RUL) has substantially increased in recent years due to the powerful data processing capabilities. However, the relationships and interdependencies of operation parameters in non-Euclidean space are ignored utilizing the current deep learning-based methods during the degradation process for engine. To address this challenge, an improved sand cat swarm optimization-assisted Graph SAmple and aggregate and gate recurrent unit (ISCSO-GraphSage-GRU) is proposed to achieve RUL prediction in this work. Firstly, the maximum information coefficient (MIC) is utilized for describing the interdependent relations of measured parameters. Building on this foundation, the constructed graph data is used as input to GraphSage-GRU so as to overcoming the shortcomings of existing deep learning methods. Additionally, this work proposed an improved sand cat swarm optimization (ISCSO) to improve the predicted performance of GraphSage-GRU, including tent mapping in population initialization and a novel adaptive approach enhance the exploration and exploitation of sand cat swarm optimization. The CMAPSS dataset is used to validate the effectiveness and advancedness of ISCSO-GraphSage-GRU, and the experimental results show that the *R*^2^ of the ISCSO-GraphSage-GRU is greater than 0.99, RMSE is less than 6.

## Introduction

Benefiting from the development of sensor technology, prediction and health management (PHM) techniques for complex systems (i.e. aero-engines) use the collected measured data as solution to improve the reliability and intelligence^1^. In aero engines, it consists of turbines, fuel/oxygen pumps, etc., are operating in complex and variable environments, which leads to complex degradation patterns. Therefore, it is essential to provide health monitoring state during operation process, based on which a prediction of the remaining useful life (RUL) can be realized, which will ensure the normal operation of the engine^2^. Currently, the RUL prediction for aero engines can be categorized into model-based and data-driven approaches. For the model-based method, it is designed to achieve the RUL prediction by constructing an accurate simulation model based on the physical and mathematical mechanism from engine. However, it is difficult to construct an accurate physical model for complex systems due to the diversity of measured data^3^. In recent years, the data-driven RUL prediction approach have shown a lot of promise. Some shallow machine learning such as random forest^4^, extreme learning machine^5^, gradient boosting decision tree^6^, etc. and some deep learning methods like convolutional neural network (CNN)^7^, recurrent neural network (RNN), have been successfully applied to the RUL prediction. Notably, the shallow machine learning methods rely heavily on human-defined features, which may obtain an invalid predicted results^8^. In constant, it is possible for deep learning methods to achieve adaptive feature extraction, which avoids the influence of human experience^9–11^. Among the deep learning methods, the historical information and current information can be used by RNN due to the particular network structure. Gated recurrent unit (GRU), a variation of RNN, is better at handling long sequences by incorporating several gates to control the memories in the model^12^. For example, Zhang et al.^13^ used GRU to obtain implicit degradation information from sensors based on domain knowledge and achieved accurate RUL prediction. Zhou et al.^14^ proposed an improved GRU to mitigate the forgetting rate, which is applied in RUL prediction. These studies have shown that the GRU have shown a lot of promise for RUL prediction.

Although it is possible for GRU to realize the RUL prediction of the engine, the co-dependence between the degradation data in the non-Euclidean space is ignored. Specifically, GRU exploit the potential relationships among different operation parameters of engines in a predefined order, ignoring the arbitrary interdependencies between data or various physical measurements of multiple sensors^15^. Recently, graph neural networks (GNNs) have shown potential in the field of aero-engine RUL prediction task^16^. This kind of methods utilize the propagation method on all nodes overlooking the order of the nodes and update the weights of aggregated neighborhood nodes, Providing the possibility for exploring arbitrary interdependencies between data or various physical measurements of multiple sensors in engines^17^. Indeed, GNNs have been successfully utilized in traffic fields, materials, etc. For example, Kong et al.^18^ verified the predicted performance of GNN with real traffic data. Reiser et al.^19^ introduced and summarized a roadmap for the potential and application of graph neural networks in the field of chemistry and materials. In^20^, GNNs were introduced to analysis the operation relationship of bearings by Yang et al. However, GNNs have some drawbacks such as high computational complexity and memory consumption, leading to limitations in computational efficiency and operational memory when dealing with engine degradation data^21^. As a variant of GNN, the computational efficiency and over smoothing problem of which is mitigated with GraphSAGE by sampling and aggregating neighbouring nodes. In^22^, GraphSAGE was used to achieve the traffic prediction so that both the dynamic spatial and temporal dependencies could be captured. Chen et al.^23^ used air-conditioning operation data and GraphSAGE to build a prediction model for air-conditioning energy consumption, and accurate energy consumption prediction was achieved. Zhu et al.^24^ used GraphSAGE to achieve the bearing fault diagnosis, which indicated that GraphSAGE have potential and application prospects intelligent diagnostic field. These studies show that GraphSAGE will hold great promise to pave the way on prognostics for the engineering data cases. However, GraphSAGE has rarely used for RUL prediction except for few results on engine.

In view of the neural network, the hyperparameters such as learning rate, number of neurons, etc. are the essential factors affecting the constructed RUL prediction model^17^. Traditional hyperparameter tuning methods depend on manual experience, leading to problems such as falling into local optimums, and inefficiency. In recent years, some swarm intelligence optimization algorithms have been introduced to alleviate these problems, such as genetic algorithm (GA)^26^, alpine skiing optimization algorithm (ASO)^27^, grey wolf optimization algorithm (GWO)^28^, Artificial Bee Colony (ABC)^29^, greylag goose optimization (GGO)^30^, puma optimizer (PO)^31^, football optimization algorithm (FbOA)^32^, liver cancer algorithm (LCA)^33^, parrot optimizer (PO)^34^, artemisinin optimization (AO)^35^, polar lights optimization (PLO)^36^, rime optimization algorithm (RIME)^37^ and so on. Although the hyperparameters of neural networks can be optimized by these optimization algorithms, local optimum, premature maturity, etc., can be caused. Inspired by these problems, the improvement of traditional optimization algorithms has been a hot research topic. As one of the state-of-the-art swarm intelligence optimization algorithms, sand cat swarm optimization algorithm (SCSO) is not only simple and easy to understand but also efficient. In constant to other algorithms, it controls the transitions in the exploration and exploitation phases in a balanced manner and performed well in finding good solutions with fewer parameters and operations for the hyperparameters of neural networks. There are many studies have demonstrated the effectiveness of SCSO for solving optimization problems^38–40^. Similar to other algorithms, sand cat swarm optimization algorithm (SCSO) faces a several challenges such as unhomogeneous random initialization, poor level of post-development, etc^41^. Hence, it is necessary to make some improvements in SCSO to improve the global search capabilities in the during the hyperparametric search process.

To alleviate the above dilemmas, an improved RUL prediction method is proposed in this paper. The proposed method has the following three major contributions:

1. The maximum information coefficient (MIC) is introduced to the relations among measured parameters. Building on this foundation, GraphSAGE-GRU is proposed to capture the degradation information for engine to construct an accurate RUL prediction model.
2. An improved sand cat swarm optimization algorithm (ISCSO) is proposed, which includes tent mapping in population initialization and a novel adaptive approach enhance the exploration and exploitation of sand cat swarm optimization. These are used to alleviate the problems such as unhomogeneous random initialization, poor level of post-development during the hyperparameter searching process.
3. A novel RUL prediction method based on the developed GraphSAGE-GRU and ISCSO is proposed for solving the problem of acquisition of degradation information from euclidean and non-euclidean spaces. The experiments show that it has a better predictive ability.

Four sections follow this introduction. The related theories are described in Preliminary section. The proposed RUL prediction model and the ISCSO are presented in Proposed method section. The CMAPSS dataset is utilized to verify the effectiveness of the proposed algorithm in Results and discussion section. Some conclusions and future work are given in “Conclusion” section.

## Preliminary

### GraphSAGE

Traditional neural networks can only handle regular relations in Euclidean space, GraphSAGE is able to provide additional relationships and data interdependencies^42^. In the GraphSAGE, the computational efficiency and memory problems have been improved compared to GCN. Specifically, it is constructed by sampling the neighboring nodes of the graph data in the degradation samples to generate embedded representations of the nodes. In this way, the representation of the engine’s degradation information in non-Euclidean space can be realized. Similar to graph neural networks, the constructed degradation sample can be represented as:

1 $$G=(V,E,A)$$

where *V* and *E* denote the nodes set and edge in graph samples. *A* is the adjacency matrix, which is utilized to represent the weights of any two nodes.

**Fig. 1:**
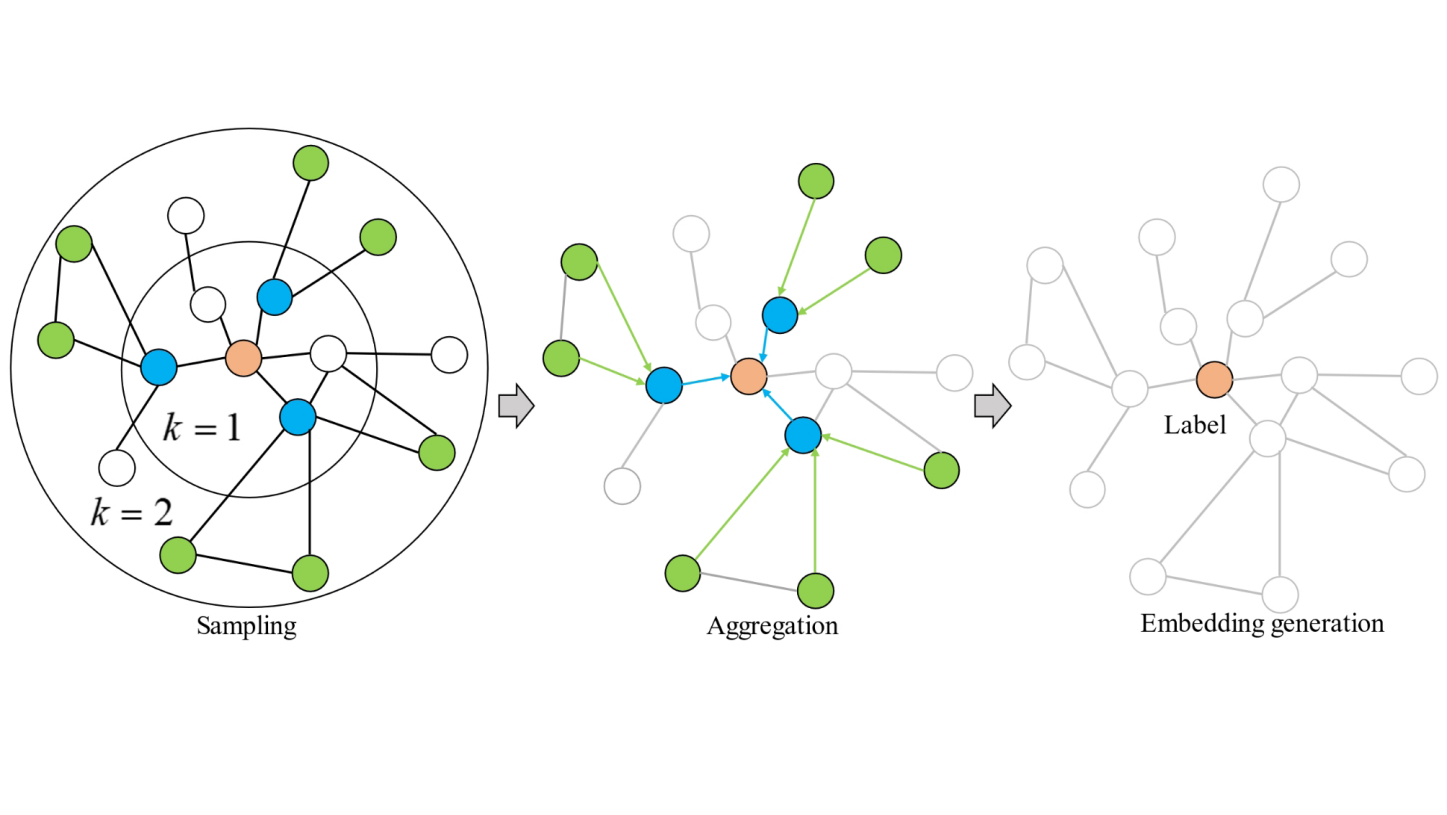
The process of graphSAGE.

Then, the constructed graph data are fed into the GraphSAGE to. Firstly, GraphSAGE can realize fixed-size sampling for the neighboring nodes of each graph node. On this basis, the aggregation function (e.g. mean, sum, LSTM, etc.) is used to realize the representation in the next layer from previous layer. The advantages of this is that the Computational efficiency and generalization of GCN are improved. The aggregation process is shown in Fig. 1.

2 $$h_{{N(a)}}^{l}=aggregate(\{ h_{b}^{{l - 1}}\} )$$

3 $$h_{a}^{l}=\sigma (W\cdot concat(h_{a}^{{l - 1}},h_{{N(a)}}^{l}))$$

4 $$h_{a}^{l}=\sigma ({W^k}\cdot MEAN(\{ h_{a}^{{l - 1}}\} \cup \{ h_{{N(a)}}^{l},\forall b \in N(a)\} ))$$

5 $$h_{{a1}}^{l}=\frac{{h_{a}^{l}}}{{||h_{a}^{l}||{}_{2}}}$$

where $h_{a}^{{(l)}}$ and $h_{a}^{{(l - 1)}}$ represent the relation representation results of *a* at *l* layer and *l-*1 layer, respectively.$aggregate(\cdot )$ denotes the aggregation function, which aims at concatenating the target node with the neighboring nodes at the *l*-1 layer.$\sigma (\cdot )$ denotes the activation function.$N(a)$ is the set of all nodes. *W* and ${W^k}$ are the learnable matrix. $h_{{a1}}^{l}$ indicates the aggregation results. $concat(\cdot )$ is recognized as the concatenate function.

###  Gated recurrent unit (GRU)

**Fig. 2:**
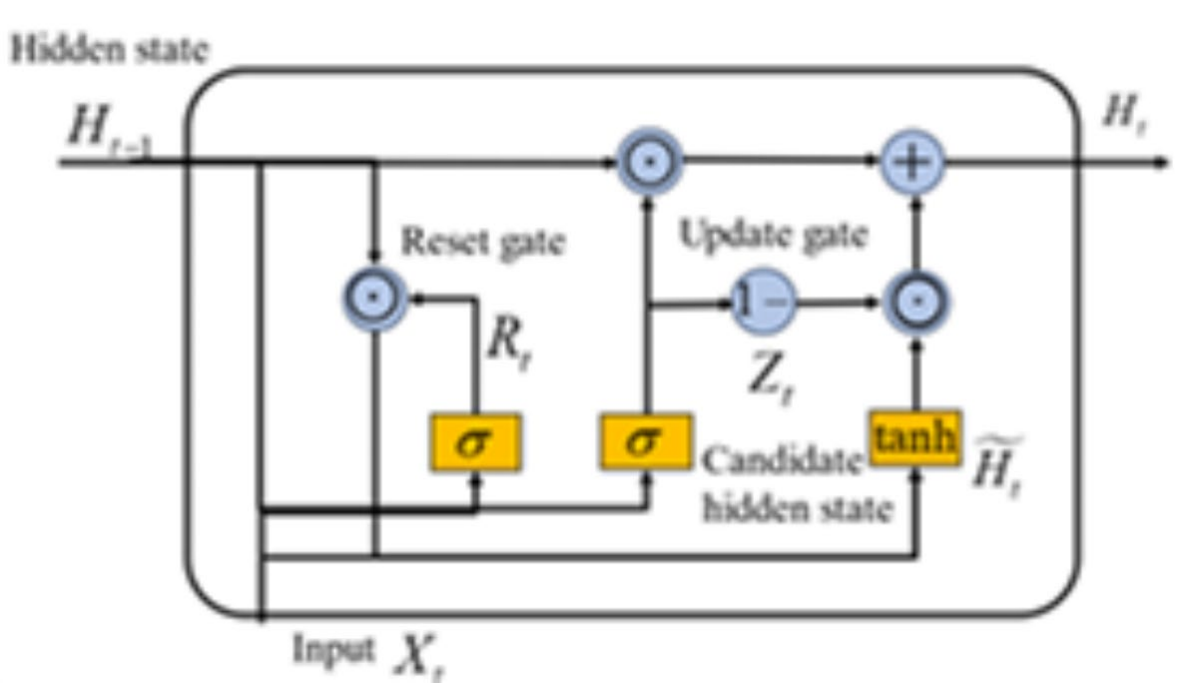
The structure of GRU.

Considering that the degradation information from the engine can be recognized as time series data, which will show cyclical and trending patterns during the degradation process, thus, time relationship is an essential feature need to be considered. Although recurrent neural network (RNN) can be utilized to obtain the degenerate hidden relationships, it is not be directly employed due to the gradients vanishing as a variant of RNN, gated recurrent unit (GRU) is an improved network to alleviate the problem of gradients vanishing in traditional RNN. Compared to long short-term memory networks (LSTM)^43^, GRU has a simpler structure and faster convergence speed, which can significantly improve the training efficiency. Its structure is shown in Fig. 2. From this figure, it can be seen that the GRU includes reset gates and update gates to control the flow and memorization of historical information. the update gate determines how much historical information is introduced in the current state. The reset gate controls the current input information based on the historical state. Namely, the larger the value of the reset gate is, the more information is added into the current state. For a single GRU structure, the output hidden state of the GRU at moment t can be represented as.

6 $${z_t}=\sigma ({W_z}[{x_t},{h_{t - 1}}]+{b_z})$$

7 $${r_t}=\sigma ({W_r}[{x_t},{h_{t - 1}}]+{b_r})$$

8 $$\widetilde {{{h_t}}}=\tanh ({W_h}[{x_t},{r_t} \odot {h_{t - 1}}]+{b_h})$$

9 $${h_t}=(1 - {z_t}) \odot {h_{t - 1}}+{z_t} \odot \widetilde {{{h_t}}}$$

where ${h_{t - 1}}$, ${h_t}$, and $\widetilde {{{h_t}}}$represent the hidden state at previous moment, hidden state at current moment and Intermediate hidden state, respectively. ${W_z}$, ${W_r}$ and ${W_h}$ are the learnable matrices, respectively. ${b_z}$, ${b_r}$ and ${b_h}$ denote the bias matrices. ${r_t}$ and ${z_t}$ indicate the output results of reset gate and update gate. $\odot$ represents the dot product.

###  Sand cat swarm optimization algorithm (SCSO)

Considering the fact that hyperparameters such as filtered redundant information, number of hidden layers, and number of layers of neural network can affect influence the RUL prediction performance, it is necessary to adjust the hyperparameters according to the input degraded data in order to obtain an effective RUL prediction model. Traditional hyperparameter optimisation methods are either experience- or time-consuming, which cannot guarantee the best parameter combination. As a swarm intelligence optimization algorithm, Sand Cat Swarm Optimization Algorithm is proposed inspired by the behaviour of sand cats, for the detection of low-frequency noises, sand cats are able to locate their prey both above and below the ground with its unique ability. The basic setup of the algorithm can be represented as

10 $${r_G}={S_M} - (\frac{{{S_M}*t}}{{{t_{\hbox{max} }}}})$$

11 $$R=2*{r_G}*rand - {r_G}$$

12 $$r={r_G}*rand$$

where ${S_M}$ is set to 2, *t* and *t*_*max*_ represent the iteration number at current moment and the max iteration.${r_G}$ is decreased from 2 to 0 as the number of iterations increases. *R* is the parameter for choosing in the searching process and attack process. Notably, the sand cat enters the attack state when |R|≤1, otherwise it is the search state. *r* denotes the sensitivity range of each sand cat. rand is a random number between 0 and 1. The mathematical model of the attack is represented as follows

13 $${P_r}=|rand*P_{{bc}}^{t} - P_{c}^{t}|$$

14 $$P_{c}^{{t+1}}=P_{c}^{t} - r*{P_r}*\cos (\theta )$$

where ${P_r}$ denotes a random position around the optimal position, which is used to ensure that the sand cat can approach the optimal position. $P_{c}^{{t+1}}$ represent the updated location. $\theta$ is chosen randomly by roulette to avoid falling into a local optimum. When |R|>1, the sand cat searches for prey locations within its sensitivity range, and the position can be expressed as

15 $$p_{c}^{{t+1}}=r*(p_{b}^{t} - p_{c}^{t}*rand)$$

where $p_{b}^{t}$ denotes the optimal candidate position.

## Proposed method

The engine can be considered as a complex equipment, in which the measured data is characterized by large scale, non-linearity and high dimensionality. In this work, a RUL prediction method for complex equipment based on GraphSAGE and GRU is proposed.

### Adjacency matrix for graphsage

For the engine, *V* is organized by the sensors in the engine such as temperature, rotational speed, etc., which is determined by a fixed sliding time window. Specifically, the length of each window and its stride is set to *T* and *S*, respectively. Each input sample *V* of engines is recorded as *x*_*i*_. *x*_*i*_ is a sub-matrix *X* of $T * N$. *N* represents a parameter such as temperature, coulomb efficiency, etc., in one operation cycle. The parameters inside the *x*_*i*_ represent the condition of the engines over time. This allows the degradation trends in engines to be preserved. *E* is composed of nonlinear relationships between different parameters at different time within a sliding time window. Notably, the nonlinear relations of these sensors are difficult to be captured using mathematical equations. Thus, the maximum information coefficient (MIC) is introduced to describe the nonlinear relations among the nodes^44^. Take the temperature and the speed at same time as an example, the function is defined as follows:

16 $$I([P,S],x,y)=\int {p(x,y){{\log }_2}\frac{{p(x,y)}}{{p(x)p(y)}}dxdy}$$

where $p(x,y)$ denotes the joint probability, $p(x),p(y)$ represent marginal probability distribution. $MIC([P,S])$ can be expressed as

17 $$MIC([P,S])=\mathop {\hbox{max} }\limits_{{ab<B}} \frac{{\hbox{max} I\{ [P,S],x,y\} }}{{{{\log }_2}\hbox{min} \{ a,b\} }}$$

where *a*, *b* denote the number of x-axis and y-axis grids, respectively. Mutual information values can be computed on different grids, and *B* represents the upper limit of the grid. $MIC([P,S])$ is recognized as the attributes of node rotational speed and pressure. The other parameters are similar as well. Notably, different edges of the same sensor are regarded as the interactions from other sensors, some edges reflect the collaboration between sensors, and there are some edges indicate disturbances during operation. In order to effectively represent these connections between sensors, it is necessary to filter redundant information while reducing information loss. It will improve the prediction precision of the RUL prediction model and the interpretability. Therefore, filtering selection method is used to select the feature edges set.

### An improved sand cat swarm optimization algorithm based on multi-strategy (ISCSO)

Population initialization is an essential part of the swarm intelligence optimization algorithm. For example, a fuller coverage of the solution space can be provided by the uniform distribution rather than the random distribution. In the traditional SCSO, random distribution was used to achieve the population initialization, which is difficult to cover the entire solution space. In contrast, chaotic sequence in the solution space has the characteristics of ergodicity, randomness and regularity, which can find the search space with a higher probability than random search. In order to get better initial solution, Tent mapping is introduced to improve the solution space. Notably, Tent mapping suffer from problems easily on small loop cycles and immobile points, and the optimal solution can only be found when the optimal solution is only edge-valued. Thus, an improved Tent mapping with beta-distributed random numbers is introduced, which is expressed as^45^

18 $$y_{i}^{{j+1}}=\left\{ \begin{gathered} \mu \times y_{i}^{j}+\delta \cdot {b_{betarnd}}(q,m),y_{i}^{j}<0.5 \hfill \\ \mu \times (1 - y_{i}^{j})+\delta \cdot {b_{betarnd}}(q,m),y_{i}^{j} \geqslant 0.5 \hfill \\ \end{gathered} \right.$$

where $y_{i}^{j}$ and $y_{i}^{{j+1}}$ denote the j-th dimensional and the (*j* + 1)-th dimensional component of the *i*-th soldier. $\mu$ represents the chaos coefficient, which is defined as 2. ${b_{betarnd}}$ is the random numbers of the beta distribution. $\delta$ is the shrinkage factor. $q,m$ are the parameters of the beta distribution. $\delta$, *q* and *m* are set to 0.1,3 and 4. On this basis, the positional variables of the initial population individuals are defined as

19 $$Z_{i}^{j}=w{c_j}+y_{i}^{j} \times (u{c_j} - w{c_j})$$

where $w{c_j}$ and $u{c_j}$ denote the lower and upper boundaries of hyperparameters at the j-th dimension.

Similar to other optimization algorithms, it is inevitable that SCSO suffers from slow convergence and easily falls into the localization. Thus, an adaptive decay function is proposed to improve the global search ability of SCSO during the searching process.

20 $$E=\nu \times {e^{( - \lambda \times t)\cdot \cos (\varphi \times t+\pi )}}$$

where $\lambda$ is considered as the decay rate, which is set at 0.05. *v* is the random number in [0,2], *t* is the current iteration. $\varphi$ denotes the angular frequency, which is set to $2\pi$. Therefore, the Eq. (14) and Eq. (15) are redefined as:

21 $${P_r}=|rand\cdot P_{{bc}}^{t} - P_{c}^{t}|$$

22 $$P_{c}^{{t+1}}=P_{c}^{t} - r\cdot {P_r}\cdot \cos (\theta )\cdot E$$

23 $$p_{c}^{{t+1}}=r\cdot (p_{b}^{t} - p_{c}^{t}\cdot rand\cdot E)$$

**Fig. 3:**
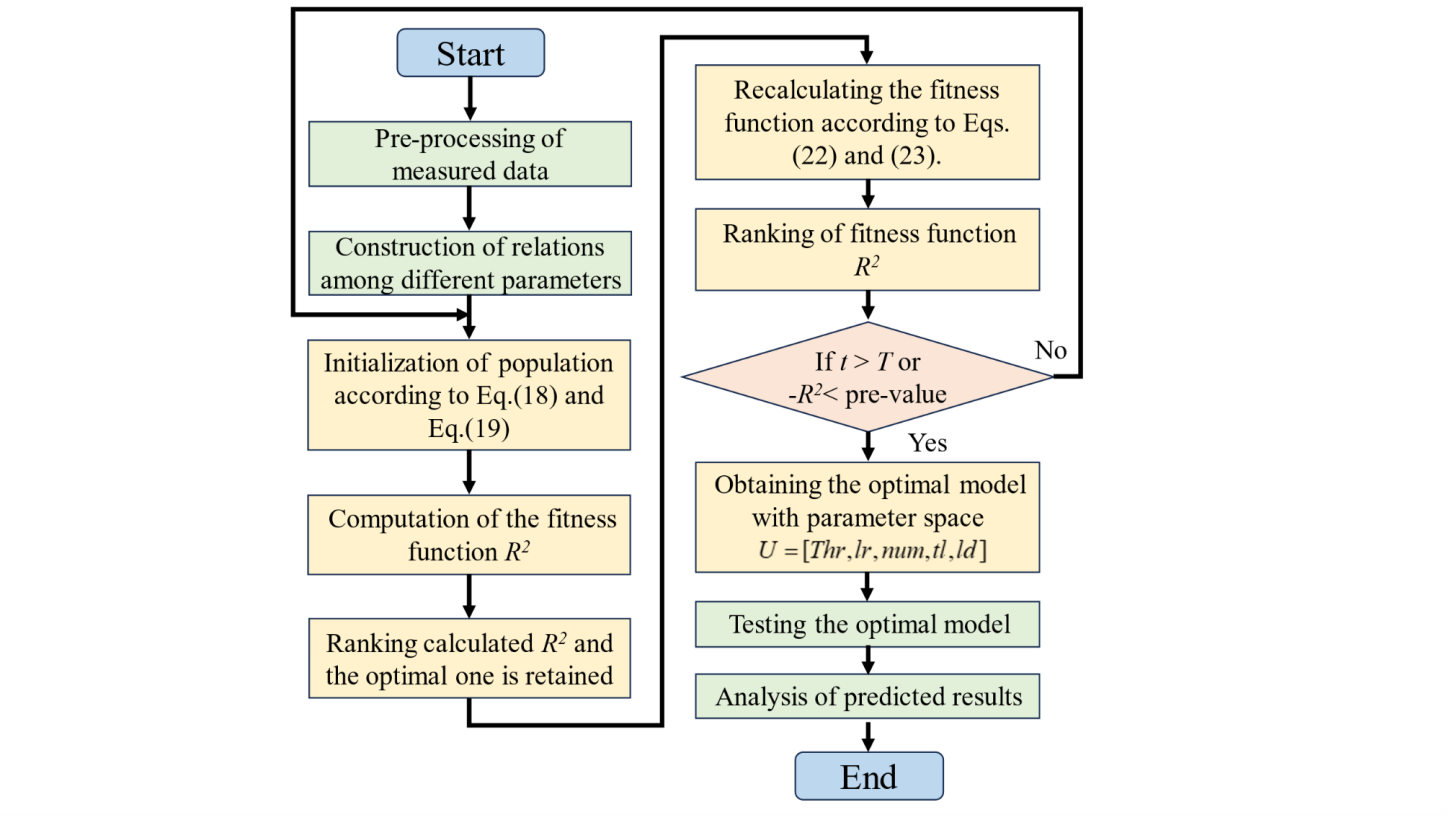
The flowchart of RUL prediction based on ISCSO-GraphSAGE-GRU method.

The Fig. 3 is the flow chart of ISCSO being used for hyperparameter optimization in GraphSAGE-GRU. From this Figure, it can be seen that the hyperparameters need to be optimized include the learning rate, the number of hidden layer for GRU, the number of neurons for GraphSAGE and GRU. The detailed workflow is illustrated as follows:

In the engineering problems, sliding window technique is used to obtained the local degradation relationship for engines. Then, the maximum information coefficient (MIC) is introduced to describe the nonlinear relations for different measured parameters for a sliding window. Considering that the data scales for engines are different, leading to the analytical bias, data fusion bias, etc. Thus, it is essential to make normalization of the measured data. In this work, min-max normalization is employed to implement the normalization for training datasets.

24 $${x_{norm}}=\frac{{{x_i} - {x_{\hbox{min} }}}}{{{x_{\hbox{max} }} - {x_{\hbox{min} }}}}$$

where ${x_{norm}}$, ${x_{\hbox{max} }}$ and ${x_{\hbox{min} }}$ denote the normalized data, the maximum value and the minimum value of the current measured parameter. Then, the normalized data are fed into the GraphSAGE-GRU, and the ISCSO is used to the optimization of hyperparameters for GraphSAGE-GRU. The detail are as follows:

*Step 1*: Initialize the parameters of ISCSO. The size of soldiers population *U* is set to *P*, the maximum iteration is given by T. The hyperparameters in the *U* is defined as follows:

25 $$U=[lr,n{h_G},n{h_g},nl]$$

where $lr$ represents the learning rate for GraphSAGE-GRU, $n{h_G}$ and $n{h_g}$ denote the number of neurons for GraphSAGE and GRU. $nl$ is the layer of GRU.

*Step 2*: Calculate the fitness function, a series of trained models are obtained by training set based on *U.* Then, the *R*^*2*^ from these trained models are used as the fitness function and the hyperparameters set are sorted according to the *R*^*2*^ while the optimal information for the current time is retained.

*Step 3*: Calculating the hyperparameters set according to Eq. (22) and Eq. (23). Building on this foundation, Calculating the *R*^*2*^ and replace the current soldiers if it is better than the current soldiers.

*Step 4*: The optimal parameters of the optimal GraphSAGE-GRU and the optimal model are outputted if the maximum iteration is reached or the threshold for *R*^*2*^ is satisfied, otherwise execute 1–4.

Finally, the testing set is used to predict the RUL based on optimal GraphSAGE-GRU. The number of soldiers and the max iteration are 5, 100, respectively. Besides, the weight of GraphSAGE-GRU and bias are updated by Adam and the number of training epochs is set to 100.

###  RUL prediction method based on ISCSO-GraphSAGE-GRU

**Fig. 4:**
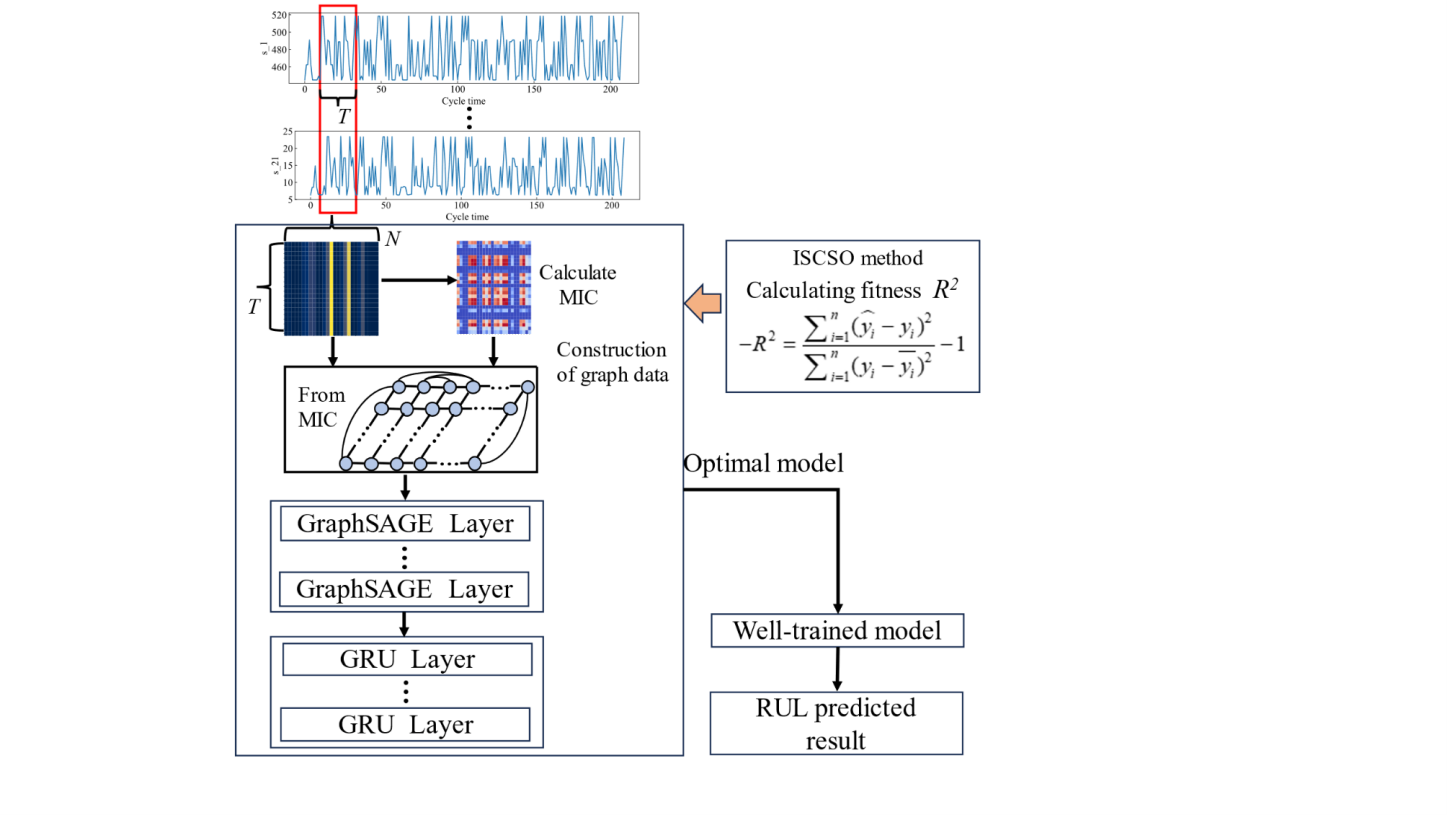
The flow of the proposed method.

The details of the proposed method are shown in Fig. 4. From this figure, it can be seen that the proposed method consists of three parts, including graph construction, acquisition of spatial-temporal degradation relations, and the optimization of hyperparameters. Firstly, fixed-size sliding window is introduced to allows the local degradation trends in engines to be preserved. Then, the maximum information coefficient (MIC) is used to describe the nonlinear relations for different measured parameters. On this basis, fixed-size sliding window is used to construct graph data. Notably, parameters at different moments within the sliding time window are used as nodes of the graph data while the MIC is used to measure the relationship between these nodes. Not all relationships between measured parameters have positive effect on the RUL prediction task. Thus, it is essential to filter out redundant edge information based on MIC value. According to the guidelines of correlation, two nodes are considered to be correlated when the normalized correlation coefficient is greater than 0.2. Besides, 30% of relationships in the filtered information are random selected, which are used to ensure diversity of information. The above information is fed into the proposed GraphSAGE-GRU so as to RUL prediction model for engines can be constructed. Moreover, considering that the RUL prediction performance can be affected by the hyperparameters of the neural network, such as the number of hidden layers, number of neurons, etc. Therefore, the ISCSO is proposed to proposed to optimize the GraphSAGE-GRU while the global search performance of SCSO is guaranteed. Finally, the optimized model is used for the RUL prediction, and the test data set is used to validate the effectiveness of the proposed method.

## Results and discussion

The structure of engines is complex, which leads to expensive maintenance costs. Therefore, accurate RUL prediction for operational status of aircraft engines is an effective and important safety precaution.

###  Data description and the evaluation metrics

**Fig. 5:**
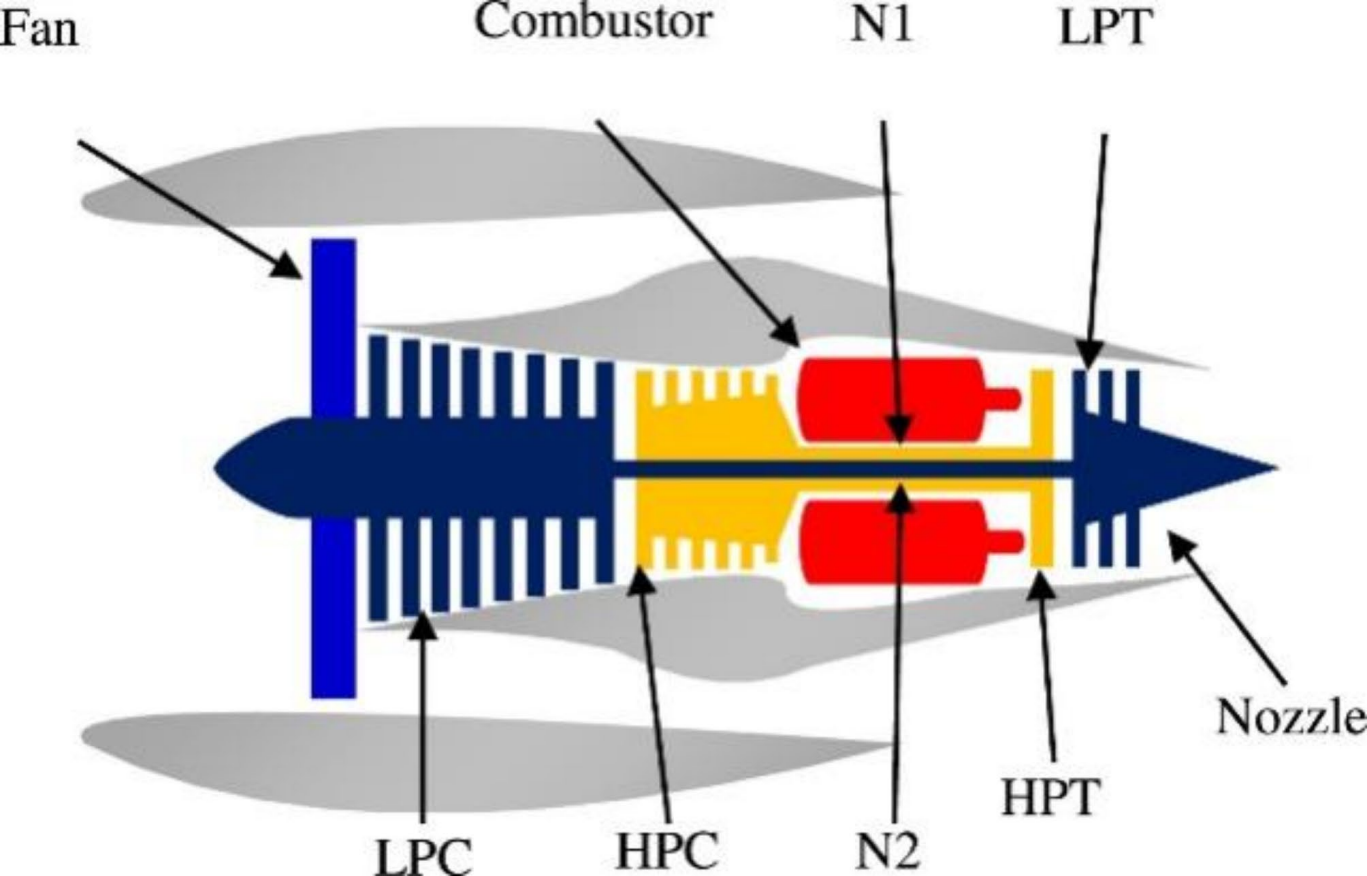
The simulated engine diagram in CMAPSS.

**Fig. 6:**
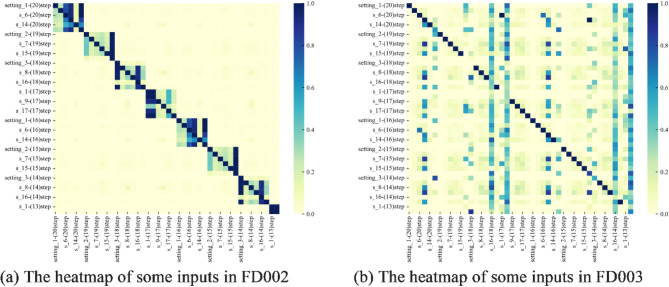
The heatmap of some inputs in a window.

The dataset used in this work is prom the commercial modular aero-propulsion system simulation (C-MAPSS)^46^. The schematic diagram of the engine is shown in Fig. 5. It consists of four sub-datasets composed of unit number, time stamp, three configurations, and 21 sensors. The details are shown in Table 1 and the description is listed in Table 2. In this work, FD002 and FD003 are used to validate the effectiveness of the proposed methodology. Inspired by literature^47^, the length of fixed-size sliding window is set to 20. And some of the MIC results for two datasets is shown in Fig. 6. From this figure, it is observed that a large number of normalized MICs are less than 0.2, which can be considered as very weakly correlated. Thus, the MIC threshold is set to 0.2 in this work is reasonable. Notably, it also can be seen that the autocorrelation of some parameters is very weak, caused by the limitations of the MIC^48^. This is considered as future work that can extract more degradation information.

**Table 1:** The details of CMAPSS.

Dataset	FD001	FD002	FD003	FD004
No. of engines in training dataset	100	260	100	249
No. of engines in test dataset	100	259	100	248
Training trajectories	17,731	48,558	21,120	56,815
Testing trajectories	10,696	29,070	13,696	37,742
Max/min cycle for train	362/128	378/128	525/145	543/128
Max/min cycle for test	303/31	367/21	475/38	486/19
Operation Status	1	6	1	6
Faults mode	HPC degradation	HPC degradation	HPC degradation Fan degradation	HPC degradation Fan degradation

**Table 2:** Output parameters C-MAPSS turbofan engine datasets.

Sener	Parameter	Description
1	T2	Total Temperature in fan inlet (oR)
2	T24	Total Temperature at LPC outlet (oR)
3	T30	Total Temperature at HPC outlet (oR)
4	T50	Total Temperature at LPT outlet (oR)
5	P2	Pressure at fan inlet (psia)
6	P15	Total pressure in bypass-duct (psia)
7	P30	Total pressure at HPC outlet (psia)
8	Nf	Physical fan speed (rpm)
9	Nc	Physical core speed (rpm)
10	Epr	Engine pressure ratio (—)
11	Ps30	Static pressure at HPC outlet (psia)
12	Phi	Ratio of fuel flow to Ps30 (psi)
13	NRf	Corrected fan speed (rpm)
14	Nrc	Corrected core speed (rpm)
15	BPR	Bypass ratio (—)
16	farB	Burner fuel air ratio (—)
17	htBleed	Bleed enthalpy (—)
18	NF-dmd	Demanded fan speed (rpm)
19	PCNR-dmd	Demanded corrected fan speed (rpm)
20	W31	HPT coolant bleed (Ibm/s)
21	W32	LPT coolant bleed (Ibm/s)

Moreover, three evaluation metrics are introduced to analyze the predicted performance, including r-square (*R*^*2*^), Root mean square error (RMSE), mean absolute percentage error (MAPE).

26 $${R^2}=1 - \frac{{{{\sum\nolimits_{{m=1}}^{M} {(y_{m}^{\prime } - {y_m})} }^2}}}{{{{\sum\nolimits_{{m=1}}^{M} {(y_{m}^{\prime } - \overline {y} )} }^2}}}$$

27 $$RMSE=\sqrt {\frac{1}{M}{{\sum\limits_{{m=1}}^{M} {(y_{m}^{\prime } - {y_m})} }^2}}$$

28 $$SMAPE=\frac{1}{M}\sum\limits_{{m=1}}^{M} {\frac{{2 \times |{y_m} - y_{m}^{\prime }|}}{{|y_{m}^{\prime }|+|{y_m}|}}}$$

where $y_{m}^{\prime }$, ${y_m}$ represent the predicted RUL values and true RUL values at the *m*-th degradation samples, Notably, all the experiments in this work were realized on a personal computer based on Pytorch and PyTorch Geometric, with a CPU of i7-12700 H, a GPU of NVIDIA GeForceGTX 3060, and 16G of RAM.

###  The effect of different metaheuristic optimization algorithms

**Fig. 7:**
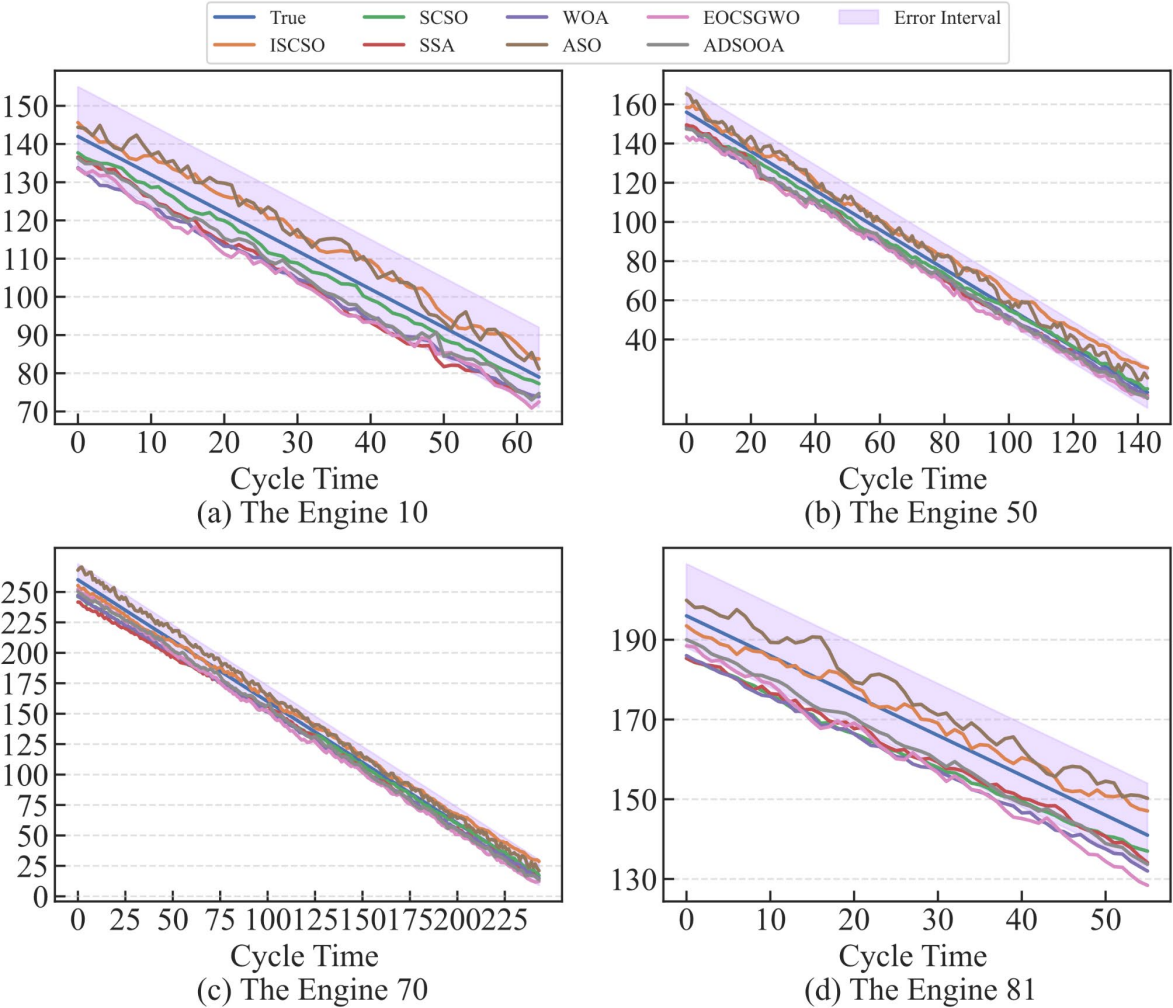
Predicted results of some engines assisted by different optimization algorithms in FD002.

**Fig. 8:**
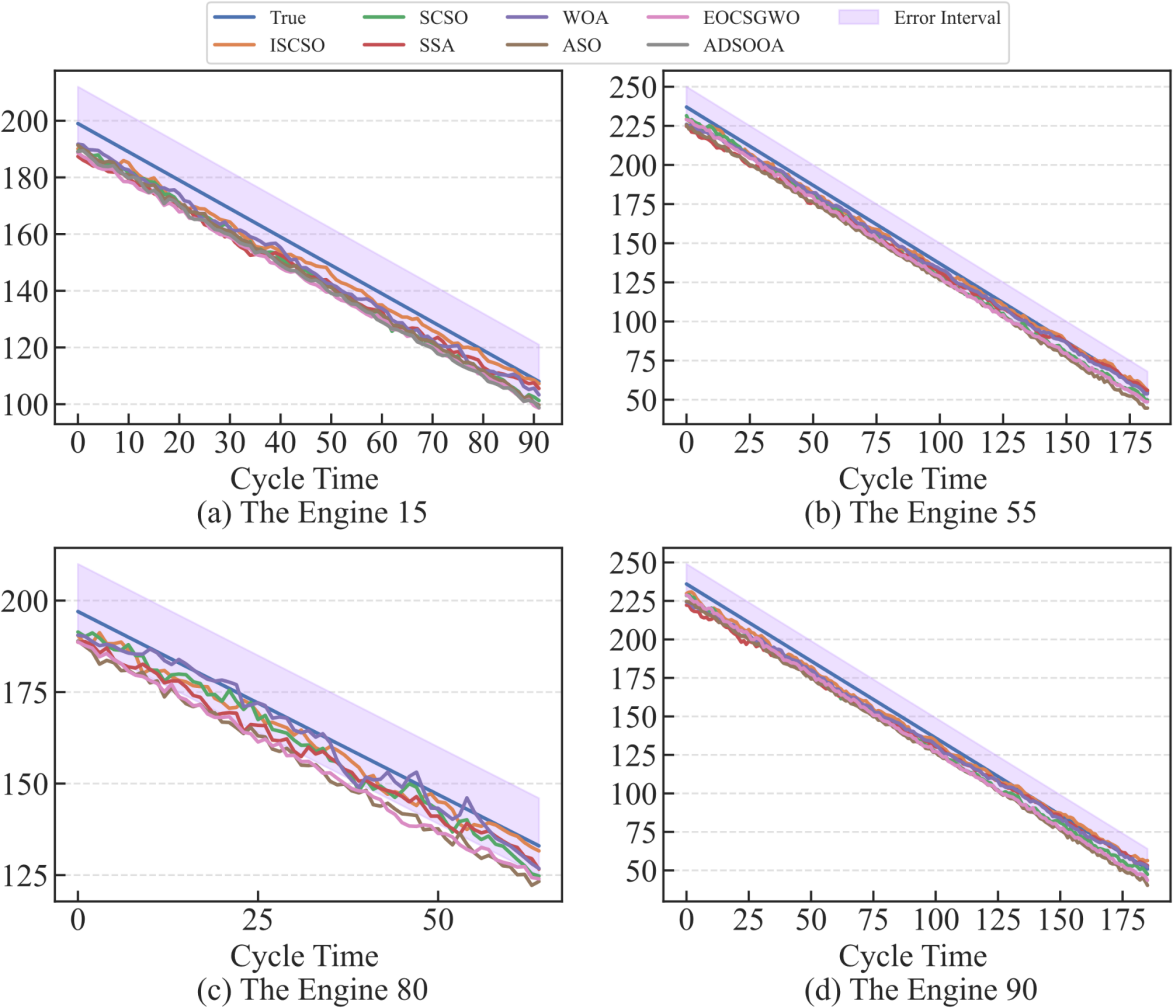
Predicted results of some engines assisted by different optimization algorithms in FD003.

In order to verify the effectiveness of the ISCSO, SCSO, sparrow search algorithm (SSA), whale optimization algorithm (WOA), alpine skiing optimization (ASO)^47^, chaotic k-best gravitational search strategy assisted grey wolf optimizer (EOCSGWO)^48^ and attack defense strategy assisted osprey optimization algorithm (ADSOOA)^28^ are firstly introduced for comparison experiment in the selected sub-datasets. The population of these methods is set to 5 and the maximum number of iterations is 100 in this experiment, and the range of hyperparameters $lr,n{h_G},n{h_g},nl$ are in the range of [0,0.001], [32,128], [32,128] and^1,4^ in two datasets. The evaluation results of these methods are shown in Tables 3 and 4. From these tables, it can be seen that the ISCSO-GraphSAGE-GRU provides more optimal predicted precision compared to other optimization algorithms. In addition, the time to obtain a stable optimal solution is also provided, which shows that the proposed method is able to reduce the running time while ensuring better prediction precision. Take the FD002 as an example, ISCSO-GraphSAGE-GRU provides the second-best results for the time to obtain a stable optimal solution. But they’re close (28.94 compared to 28.76). However, *R*^*2*^ is improved by 0.24%, RMSE and SMAPE are reduced by 11.12% and 22.85% compared to ADSOOA-GraphSAGE-GRU, respectively. These results show that ISCSO is able to alleviate the problem of falling into local optima under the same number of iterative populations on the hyperparameter optimization issue in GraphSAGE-GRU. The predicted results of some engines assisted by different optimization algorithms in FD002 and FD003 are shown in Figs. 7 and 8. Notably, error interval boundary is defined as follows^49^, in which the lower bound indicates the overprediction with 8 cycles and the upper denotes the lag prediction is 13 cycles. From these figures, it can obtain more degradation information for reliable RUL prediction so that timely maintenance for engines can be performed. Furthermore, Wilcoxon rank sum test is used to make a statistical comparison, and the p-values obtained from this are listed in Table 5. Notably, p-value is greater than 0.05 indicates that no considerable differences have been discovered between the results of the two compared optimizers. Otherwise, it is considered to be statistically huge differences between the performances of the two optimizers if the p-value is less than 0.05. From this table, it can be seen that significant differences have been discovered compared to other methods, and the ISCSO-GraphSAGE-GRU has a statistically competitive performance and achieved good and significant results.

**Table 3:** Convergence results of different optimization algorithms based on FD002.

Algorithms	*R* ^2^	RMSE	SMAPE	Time (h)
ISCSO-GraphSAGE-GRU	0.9910	5.7436	0.0368	28.94
SCSO-GraphSAGE-GRU	0.9857	7.2572	0.0432	30.47
SSA-GraphSAGE-GRU	0.9791	8.7694	0.0595	33.28
WOA-GraphSAGE-GRU	0.9804	8.4923	0.0608	34.53
ASO-GraphSAGE-GRU	0.9906	5.8799	0.0417	30.66
EOCSGWO-GraphSAGE-GRU	09771	9.1942	0.06881	32.15
ADSOOA -GraphSAGE-GRU	0.9886	6.4623	0.04779	28.76

**Table 4:** Convergence results of different optimization algorithms based on FD003.

Algorithms	*R* ^2^	RMSE	SMAPE	Time (h)
ISCSO-GraphSAGE-GRU	0.9919	7.3513	0.0327	37.23
SCSO-GraphSAGE-GRU	0.9876	9.0815	0.0526	40.07
SSA-GraphSAGE-GRU	0.9820	10.9685	0.0524	45.26
WOA-GraphSAGE-GRU	0.9862	9.6024	0.0452	46.34
ASO-GraphSAGE-GRU	0.9899	8.1916	0.0511	39.26
EOCSGWO-GraphSAGE-GRU	0.9846	10.1461	0.0643	43.16
ADSOOA -GraphSAGE-GRU	0.9895	8.3923	0.0516	36.62

**Table 5:** P-value between ISCSO-GraphSAGE-GRU and other methods in different dataset.

Algorithms	FD002	FD003
SCSO-GraphSAGE-GRU	2.91639131e-63	7.22437928e-53
SSA-GraphSAGE-GRU	2.16562708e-33	5.75721608e-22
WOA-GraphSAGE-GRU	1.21234066e-23	1.28618486e-13
ASO-GraphSAGE-GRU	2.400309e-43	4.51704877e-44
EOCSGWO-GraphSAGE-GRU	1.4343272e-21	4.85405121e-61
ADSOOA -GraphSAGE-GRU	5.93491551e-42	3.96430855e-36

### 4.3 Compared with other algorithms

**Fig. 9:**
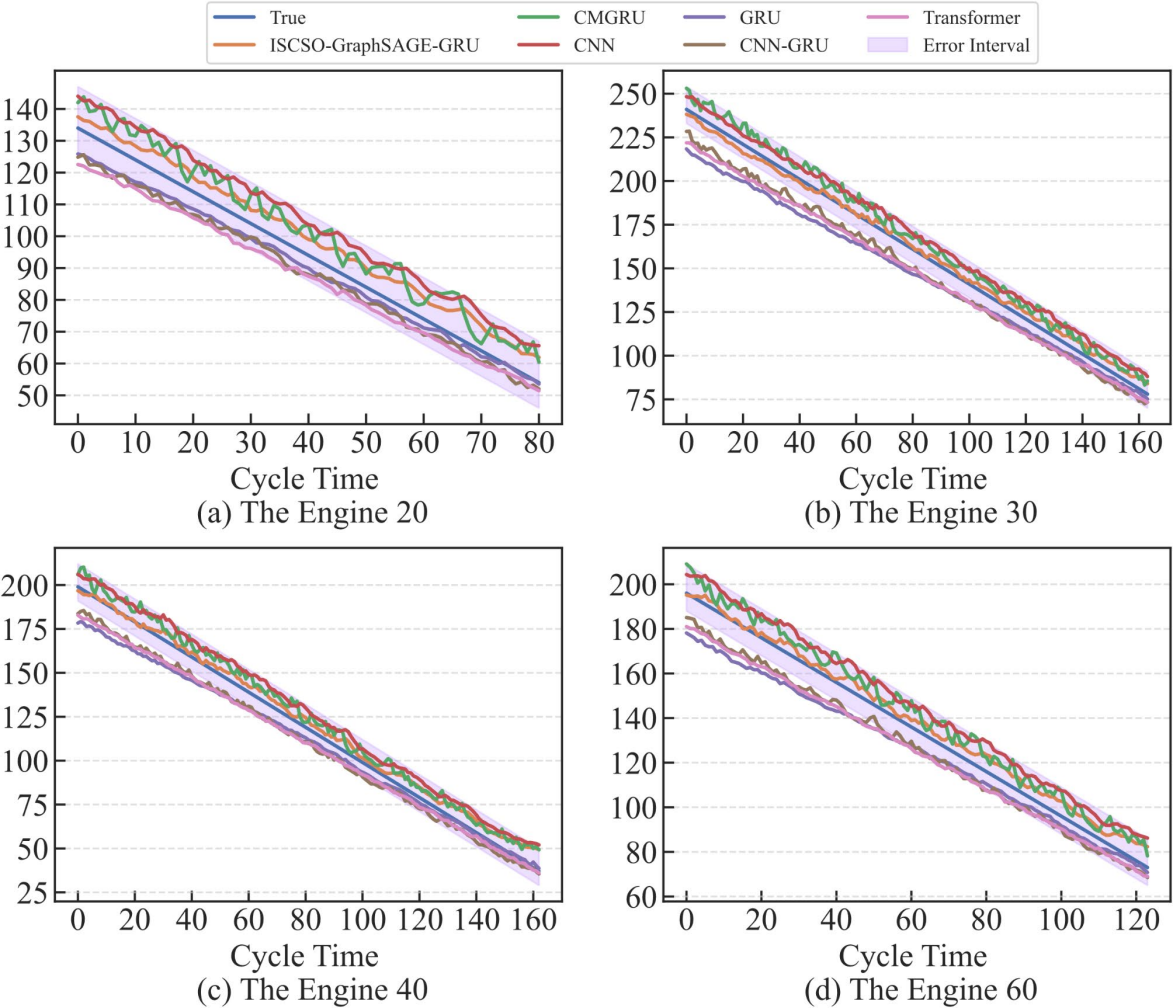
Predicted results of some engines by using different algorithms in FD002.

**Fig. 10:**
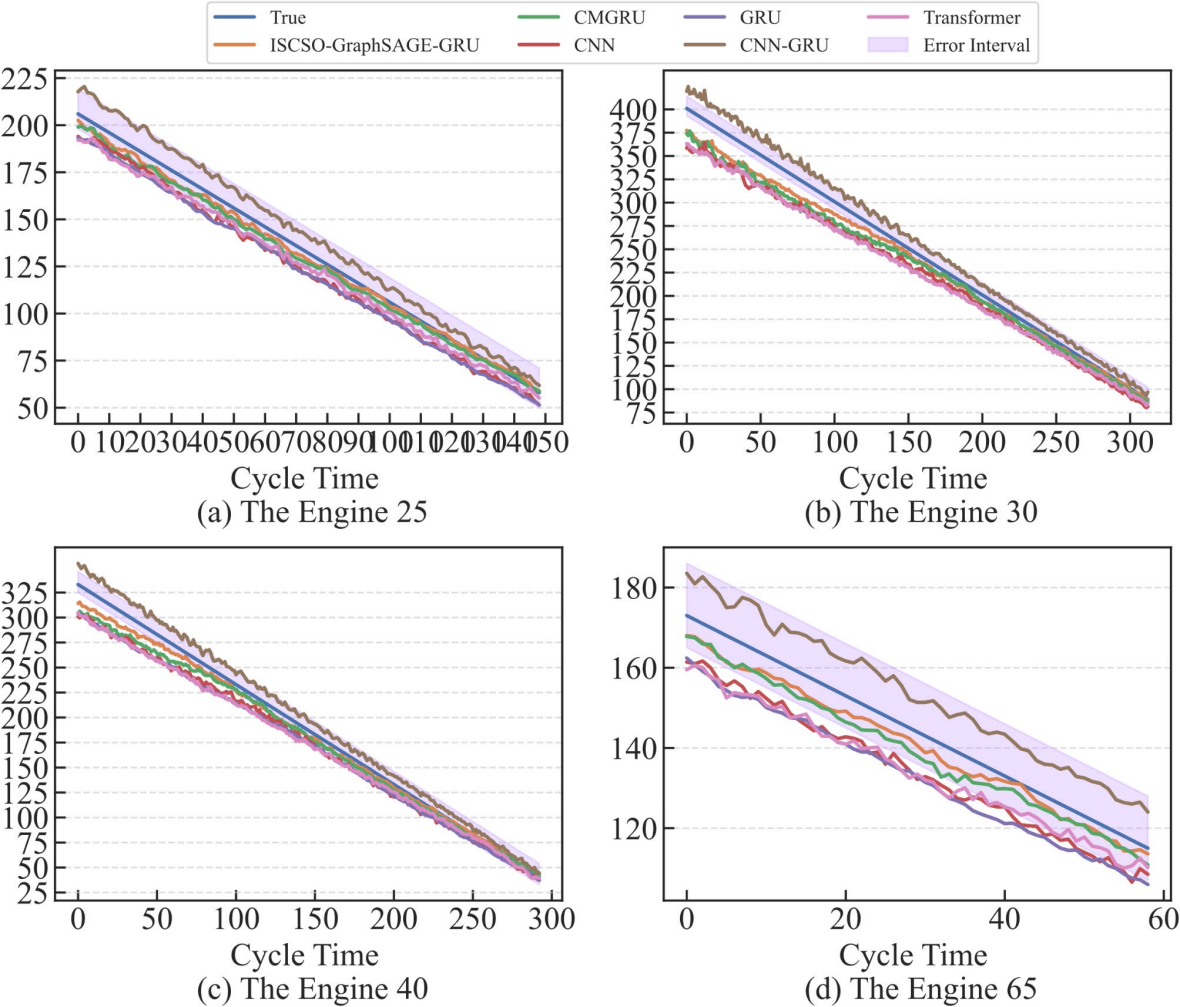
Predicted results of some engines by using different algorithms in FD003.

**Fig. 11:**
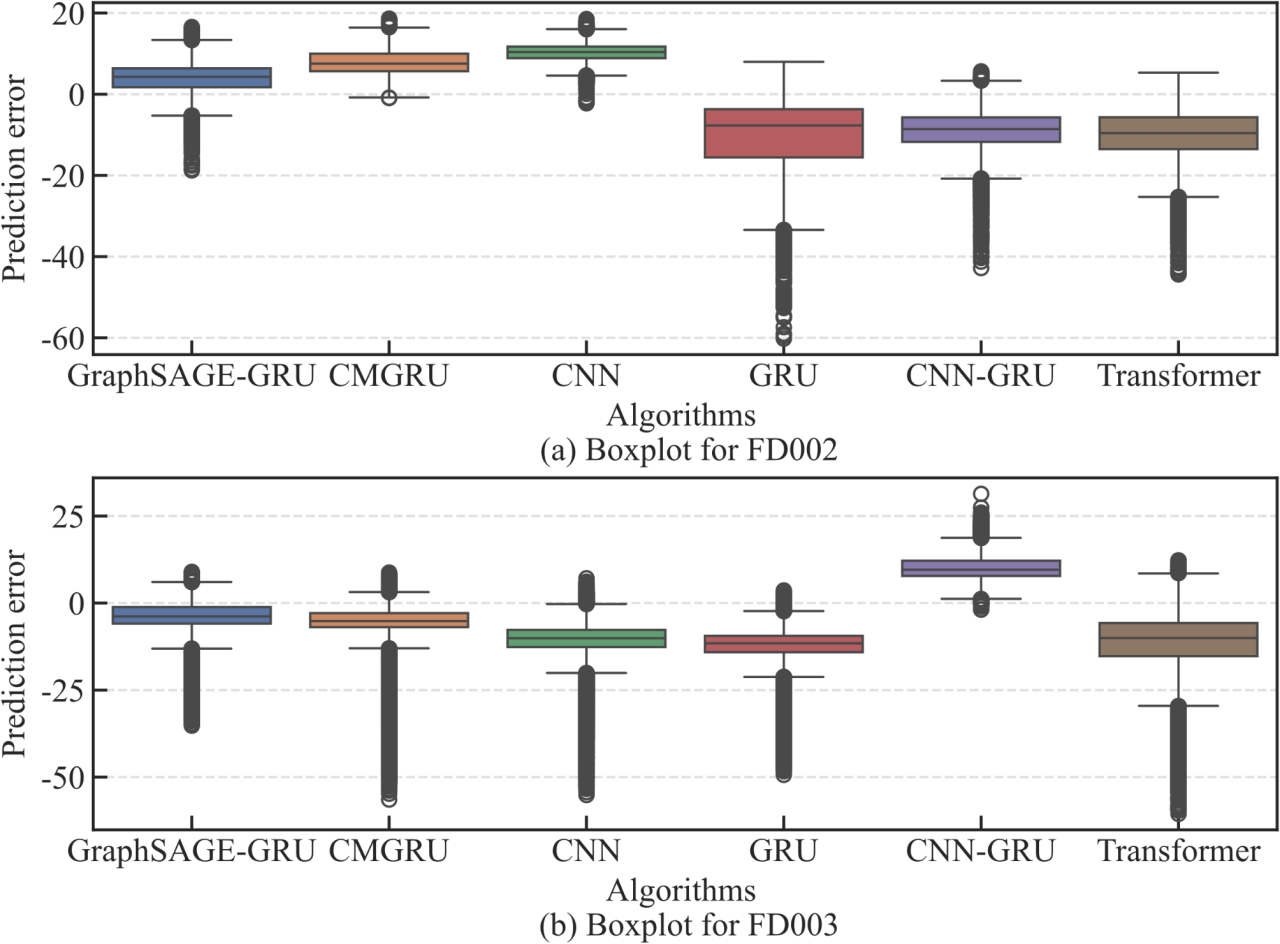
Boxplot for different algorithms in different datasets.

In order to verify the advancement of the ISCSO-GraphSAGE-GRU, some state-of-the-art algorithms are introduced, including CNN, LSTM, GRU, Consolidated Memory GRU (CMGRU), CNN-GRU and Transformer. To ensure fairness, all algorithms are optimized by using the proposed ISCSO. Tables 6 and 7 are the details of evaluation results of the engines and the predicted result of different methods can be seen in Figs. 9 and 10. Take the FD002 as an example, it can be seen that the ISCSO-GraphSAGE-GRU can provide better predicted results in constant to other methods, as listed in Table 6. For index *R*^*2*^, the result of ISCSO-GraphSAGE-GRU is higher than 0.99, meaning that it has a probability of higher than 99% to explain the reason of engine degradation, providing stable foundation for accurate prediction of the engine state. For the RMSE, the value of IMTSO-GCN is 5.7436, indicating that the predicted RUL value of IMTSO-GCN is different from the true RUL value with an average of 5.7436 cycles. The SMAPE of CMGRU is the optimal among the used traditional comparison methods. However, but value of ISCSO-GraphSAGE-GRU is still reduced by 35.55%. Among the traditional methods used in this experiment, GRU has the worst predicted performance for index *R*^*2*^, RMSE, and SMAPE. In contrast, the values of ISCSO-GraphSAGE-GRU are improved by3.67%, 54.95%, and 52.82%, which illustrates that the introduction of relationships between data interdependencies provides more potential degradation relationships, so as to fully analyse degradation information of engines. Notably, the predicted results of the different methods on FD003 are mostly superior to the ones on FD002, which is caused by the single degradation pattern of FD003 and the more complex degradation pattern of FD002. Nevertheless, ISCSO-GraphSAGE-GRU still shows competitive results, indicating that ISCSO-GraphSAGE-GRU in the real engine degradation process has shown in a large promise for RUL prediction issue. Figure 11 is the boxplot for different algorithms in different datasets. Using A as an example, the prediction errors of each cycles are within the range of -20-20, indicating that the GraphSAGE-GRU can provide competitive prediction results that enable accurate RUL prediction for engines. Moreover, the training and testing time of different RUL prediction methods are explored in FD002, including GraphSAGE-GRU, CMGRU, CNN, GRU, CNN-LSTM, and Transformer are simultaneously listed in Table 8. From this table, the GraphSAGE-GRU have a longer training time in constant to CNN and GRU. However, it has a shorter training time compared to CMGRU, CNN-LSTM and Transformer. Although the training time of GraphSAGE-GRU reaches 323.23s, it will not affect the RUL prediction because of the training is an offline process. Indeed, the training time will be greatly shortened when the hardware is improved. Also, all methods have a short testing time, which is less than 1 s. As the testing time of the proposed method is much less than the recording interval (10 s), its latency performance is sufficient for the RUL prediction in practice. In addition, in order to validate the generalization performance and effectiveness of the proposed method. PHM2010 dataset is introduced^50^. And the detailed evaluation results are listed in Table 9. From this table, it is observed that the proposed GraphSAGE-GRU could provide competitive results for *R*^*2*^, RMSE and SMAPE. The comparison results show that the advances and effectiveness of the propose method in other dataset.

**Table 6:** Convergence results of different algorithms based on FD002.

Algorithms	*R* ^2^	RMSE	SMAPE
GraphSAGE-GRU	0.9910	5.7436	0.0368
CMGRU	0.9747	9.6537	0.0571
CNN	0.9703	10.4654	0.0736
GRU	0.9559	12.7481	0.0780
CNN-GRU	0.9714	10.2499	0.0673
Transformer	0.9683	10.8129	0.0628

**Table 7:** Convergence results of different algorithms based on FD003.

Algorithms	*R* ^2^	RMSE	SMAPE
GraphSAGE-GRU	0.9919	7.3513	0.0327
CMGRU	0.9841	10.2971	0.0443
CNN	0.9707	14.1869	0.0745
GRU	0.9663	15.0237	0.0844
CNN-GRU	0.9813	11.2008	0.0708
Transformer	0.9720	13.6919	0.0791

**Table 8:** Training time and testing time of different methods in FD002.

Algorithms	GraphSAGE-GRU	CMGRU	CNN	GRU	CNN-GRU	Transformer
Training time (s)	323.23	357.57	264.84	286.12	366.58	518.47
Testing time (s)	0.4241	0.5661	0.3809	0.3924	0.5859	0.7943

**Table 9:** Convergence results of different algorithms based on PHM2010.

Algorithms	*R* ^2^	RMSE	SMAPE
GraphSAGE-GRU	0.9806	11.8182	0.0596
CMGRU	0.9783	12.5106	0.0649
CNN	0.9691	14.9208	0.0769
GRU	0.9628	16.3707	0.0875
CNN-GRU	0.9719	14.2174	0.0739
Transformer	0.9678	15.2076	0.0815

## Conclusion

In this work, a novel ISCSO-GraphSAGE-GRU is proposed to achieve the RUL prediction for engine based on the implicit relations from measured parameters in non-Euclidean spaces. The experimental results in CMAPSS dataset indicate the effectiveness of ISCSO-GraphSAGE-GRU. In the ISCSO-GraphSAGE-GRU, the maximum information coefficient (MIC) is used to illustrate the relationships for measured parameters of engines and achieve the construction of the graph data. The constructed graph samples are fed into the GraphSAGE-GRU to achieve the RUL prediction while the degradation information in non-European spaces such as the interdependence of measured parameters of engine is obtained. Moreover, the ISCSO is developed to improve the predicted performance of GraphSAGE-GRU. The experimental results on CMAPSS dataset indicate that the ISCSO-GraphSAGE-GRU can provide better predicted performance compared to traditional methods. In future, the proposed ISCSO-GraphSAGE-GRU will be extend explored, including real-time deployment with constrained computational resources, dynamic optimization and integration with digital twin systems for proactive maintenance. Specifically, the computational resources for embedded control devices in industrial scenarios are limited. Thus, it is address the feasibility of deploying ISCSO-GraphSAGE-GRU in real-time monitoring systems with constrained computational resources. In addition, explore the scalability and integrated with digital twins of the proposed method for larger datasets or other industrial domains, such as automotive or power plants is another of our research.

## Data Availability

The datasets used and/or analyzed during the current study are available from the corresponding author Ruifang Li on reasonable request via e-mail 836912005@qq.com.
